# Comparison of infant malaria incidence in districts of Maputo province, Mozambique

**DOI:** 10.1186/1475-2875-10-93

**Published:** 2011-04-17

**Authors:** Orlando P Zacarias, Peter Majlender

**Affiliations:** 1 Department of Mathematics and Informatics (DMI), Eduardo Mondlane University, Mozambique; 2 Department of Computer and System Sciences (DSV), Stockholm University, Sweden

## Abstract

**Background:**

Malaria is one of the principal health problems in Mozambique, representing 48% of total external consultations and 63% of paediatric hospital admissions in rural and general hospitals with 26.7% of total mortality. *Plasmodium falciparum *is responsible for 90% of all infections being also the species associated with most severe cases. The aim of this study was to identify zones of high malaria risk, showing their spatially and temporal pattern.

**Methods:**

Space and time Poison model for the analysis of malaria data is proposed. This model allows for the inclusion of environmental factors: rainfall, temperature and humidity as predictor variables. Modelling and inference use the fully Bayesian approach via Markov Chain Monte Carlo (MCMC) simulation techniques. The methodology is applied to analyse paediatric data arising from districts of Maputo province, Mozambique, between 2007 and 2008.

**Results:**

Malaria incidence risk is greater for children in districts of Manhiça, Matola and Magude. Rainfall and humidity are significant predictors of malaria incidence. The risk increased with rainfall (relative risk - RR: .006761, 95% interval: .001874, .01304), and humidity (RR: .049, 95% interval: .03048, .06531). Malaria incidence was found to be independent of temperature.

**Conclusions:**

The model revealed a spatial and temporal pattern of malaria incidence. These patterns were found to exhibit a stable malaria transmission in most non-coastal districts. The findings may be useful for malaria control, planning and management.

## Background

In most tropical countries, *Plasmodium falciparum *malaria has been considered a major public health problem. Around 500 million clinical malaria cases occur each year with a death toll of approximately two million people worldwide [1,2]. The charges and damages resulting from infection and death from malaria and other infectious diseases is very higher in children [3]. However, the burden that malaria brings is immeasurable [1]. Theoretically, children and adults in the region have similar risk of developing serious illness.

The largest contributions to the knowledge of malaria infection in children relate to work performed in areas of intense transmission of *P. falciparum*, as some regions of the African continent, where most children are infected in the first year of life and level of infantile mortality is high [4,5]. Meanwhile, the acquisition of immunity in the human host is an important determinant for the clinical event of *P. falciparum *malaria and, is usually influenced by the transmission profile and the individual's age [6]. Especially for developing countries like Mozambique, the task of its determination is a huge challenge given that a good number of cases of disease incidence and deaths go unreported (occur outside health centres and hospitals). This situation is even worse for rural areas, where this study was conducted. Thus, the data provided by official bodies (such as the Health Ministry, provincial departments of health, etc.) are the most reliable information to conduct impact assessments of risk and malaria incidence. Geographical patterns of malaria incidence cases are determined by factors as socio-economic status, availability and access to health centres or hospitals, including how patients seek health care facilities [7-9]. However, the temporal variation may play a role on accessibility to the hospital and transmission of malaria [9], where, for example, an increased service demand can be seen in the dry months and yet relatively few cases of malaria are recorded.

In Mozambique, malaria is endemic with a peak during wet season and most episodes occurring between January and March [10]. Given favourable conditions for transmission, epidemic malaria may also occur increasing the number of cases and deaths. Epidemic situation is usually precipitated by factors as regular rainfall and high temperatures, which increase the breeding sites of malaria vector. This paper is motivated by the analysis of malaria records for infants aged 0-4 years in a period of two years: 2007-2008 in Maputo province, Mozambique. Malaria response variable is linked to climatic explanatory covariates in space and time, with the aim to study how these factors influenced malaria incidence level in children under five years of age in the region. This will also allow comparing the levels of malaria incidence risk and proposing possible ways of intervention to health authorities. Hence, an improved description of the regional impact of malaria in infants may become very useful in designing strategies for control and management of health services as a whole. While allowing for monitoring and planning capabilities, leading to better intervention according to needs and to assist communities at high risk or even lead to further research to identify other risk factors [11]. This makes appropriate statistical models and analysis more useful and extremely important.

## Methods

### Data

**Figure 1:**
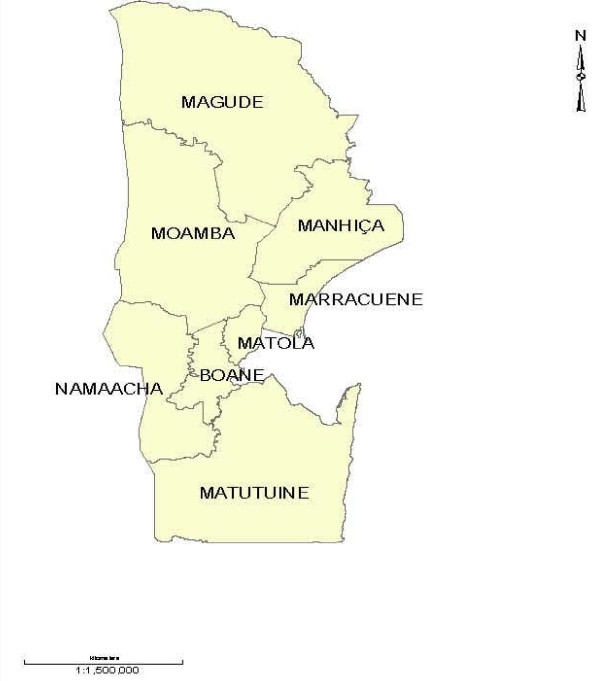
**Study region**.

The study is conducted in eight districts of Maputo province - Mozambique. The province is located in south of the country. Map of study area is given in Figure 1. Complete description of study area and available health network is given elsewhere [12].

Collected malaria data comprises records of number of episodes occurring in years 1999 to 2008, provided by provincial health directorate. After data cleaning and descriptive statistical analysis, we observed that only data for 2007 and 2008 contained complete malaria cases for children other 5 years; main group of interest in this study. While for the remaining years, the data were mixed with malaria cases of other age groups. Hence, the analysis was restricted to this time period only. Environmental data comprises rainfall, temperature (minimum, mean and maximum) and humidity, provided by National Institute of Meteorology (INAM). They are given as monthly averages forming a twenty-four long time series data. Statistical population data of the region were taken from the official 1997 and 2007 national censuses and used to compute inter-census population growth at district level for each month.

### Statistical model

To estimate the amount of spatial heterogeneity of infant malaria cases as well as associations between risk factors and infant malaria cases, Bayesian hierarchical models were designed and fitted in the presence of spatial and temporal correlations. Prior to this, a preliminary bi-variate analysis was performed in statistical package R [13]. All covariates - rainfall, temperature (minimal, mean and maximum) and humidity were found to be useful predictors of malaria incidence with significant relationship of *p < 0.001 *(Table 1).

**Table 1:** Results of bi-variate analysis of prediction variables.

Covariate	Coefficient	SE	P-value
Min-Temp	.145	.0009	< .001
Mean-Temp	.153	.0011	< .001
Max-Temp	.173	.0013	< .001
Rainfall	.0069	.00004	< .001
Humidity	.1363	.00066	< .001

The data consists of observed malaria counts *Y*_*it *_in districts *i *= 1,..., *M *and month *t *= 1,..., *T *in Maputo province. These counts are daily registered and summarized every month by district government health departments. They are then reported to provincial health directorate and shared with some other disease control programmes.

Expected cases were obtained as *E*(*i,t*) = *P*(*i,t*) * Rate where *P*(*i,t*) - is the number of children in each district *i *at a given month *t *following population projections from national census in years 1997 and 2007. The constant *Rate *is a fixed incidence rate in 10, 000 children population year [10]. The log-relative risk is modelled as,(1)

where *α *is overall incidence (intercept term), *θ*_*i *_captures the spatial variability and *ϕ*_*t *_is the monthly temporal effect. The spatio-temporal component *δ*_*it*_, captures departure from space and time main effects, which may highlight space-time clusters of risk. *β *is the vector of regression coefficients and *X*_*it *_the environmental covariates. The spatial correlation is introduced by conditional autoregressive (CAR) process,(2)

where  is the weighted average and  its variance. They are defined as  and  CAR(1) model assuming maximum spatial correlation with binary weighted matrix *ω*_*ij *_= 1 for neighbouring districts and *ω*_*ij *_= 0 otherwise, is taken. The shared interaction term *δ*_*it *_is given an exchangeable hierarchical structure, i.e.  with a constant variance [14]. The temporal effect *ϕ*_*t *_is modelled by first order random walk process *RW(1)*, where each month is influenced by variability of the previous except the first [15]. In Winbugs, *RW(1) *prior is fitted using the car.normal distribution with an appropriate specification of weight and adjacency matrices, including the vector representing the number of neighbours. Intercept term *α *and coefficients of explanatory environmental covariates *β*_*k *_for *k *= 1,...,5 were assigned uniform proper normal prior with very large variance (often used in Winbugs; MRC Biostatistics Unit, Cambridge, UK). These fixed effects are collated into the same variable γ_l _= (*α,β*) where *l *= 1,...,6 Their joint distribution is expressed as *γ *~ *MVN*(*λ*,Σ), where *λ *is the zero mean vector and Σ is the variance and co-variance *NxN *matrix. This allowed the specification of a dependency structure between coefficients *β*. To complete model definition we attribute Gamma prior for precision of all random effects, i.e. spatial, temporal and space-time component.

Parameter estimates of the model were obtained via the use of Markov chain Monte Carlo (MCMC) simulation techniques in Winbugs employing two parallel consecutive chains. A burn-in of 5, 000 iterations was first considered. This was followed by 60, 000 iterations run where the convergence of main parameters was obtained and checked. For that, we analysed Brooks, Gelman and Rubin statistics including visual examination of history and density plots. Thinning was performed as to reduce the autocorrelation level of main parameters. A thinning factor *k = 10 *was chosen. Further 60, 000 iterations were entertained to collect posterior distributions of main parameters. Monte Carlo errors (MCE) were also computed and the ratio MCE/SD found to be less than 0.05. Thus, concluded that sufficient iterations had been conducted.

## Results

A total of 60, 943 episodes of malaria were recorded in twenty-four months period. This amounts for 19% of children's population fewer than five years age for years 2007-2008, following projections of national census in 2007. Although it was not confirmed due to unavailable data, treatment-seeking behaviour in most districts may have been influenced by location of the households, financial capability and cultural related behaviour. Malaria data was analysed by initially fitting four Bayesian models following equation *(1) *above. In model 1, both space and time effects were fitted and was also included the spatio-temporal interaction. Model 2 included same parameters as model 1 except the time effect. For third model, spatio-temporal variation was left out. In model 4, the spatial random effect was not included.

The four models were compared in order to find the model with the smallest Deviance Information Criteria (DIC) value. As a result, model 4 was determined as the best fitted model (Table 2). It includes temporal effects and space-time interaction. This model has been considered for reporting results and the obtained parameter estimates are shown in Table 3. Rainfall and humidity are found to be significant predictors of malaria incidence in the region. Both show a positive association with malaria incidence. As for temperature covariates, they show a negative association. Meanwhile, every additional 1% humidity level and 1 mm rain leads to the increase of 5% and 1% on malaria incidence risk respectively. Hence, the humidity covariate seems to have apparently played higher role in malaria transmission and incidence risk for the considered demographic population group in this period. As for covariates minimum and maximum temperature, every additional degree centigrade increase reduces malaria incidence risk by 2% and 3% respectively, although these are marginally significant. While the increase of 1°C of the average temperature, does not lead to change of malaria risk incidence.

**Table 2:** Comparison of fitted models.

Model	Dbar	Dhat	pD	DIC
1- (full model)^1^	1422.24	1239.89	182.345	1604.58
2- (no time effect)^2^	1414.34	1212.05	202.287	1616.62
3-(no space-time interaction)^3^	3641.6	3590.36	51.329	3692.82
4-(non spatial effect)^4^	1402.83	1217.13	185.704	1588.53

^1^Model with spatial, temporal and space-time effects

^2^Includes space and space-time effects

^3^Includes only spatial and temporal effects

^4^Model with temporal and space-time effects

**Table 3:** Posterior estimates of intercept *α*, environmental regression coefficients *β*, temporal  and spatio-temporal  variances obtained by fitting model 4, including 95% credible intervals.

Variable	Mean	95% CI
**Intercept (a)**	-2.635	-4.081, -1.607
**Rainfall (mm)**	.006761	.001874, .01304
**Minimal Temperature (**^**o**^**C)**	-.02486	-.07359, .03632
**Maximum Temperature (**^**o**^**C)**	-.03107	-.07399, .00644
**Mean Temperature (**^**o**^**C)**	-.00132	-.0117, .008878
**Relative Humidity (%)**	.049	.03048, .06531
**Temporal variation **	.56	.2249, 1.171
**Spatio-temporal variation **	1.031	.8124, 1.304

**Figure 2:**
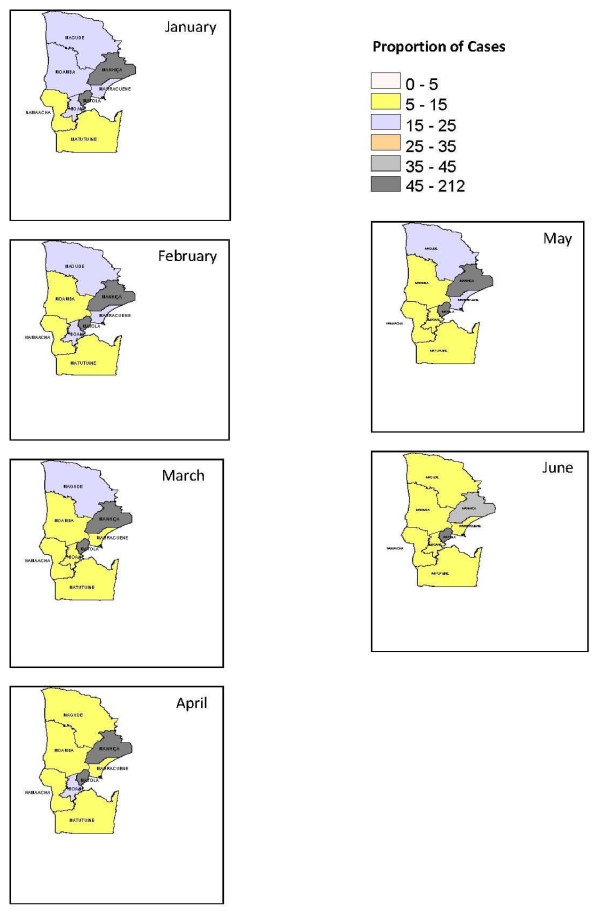
**Crude Malaria for months Jan-June**.

**Figure 3:**
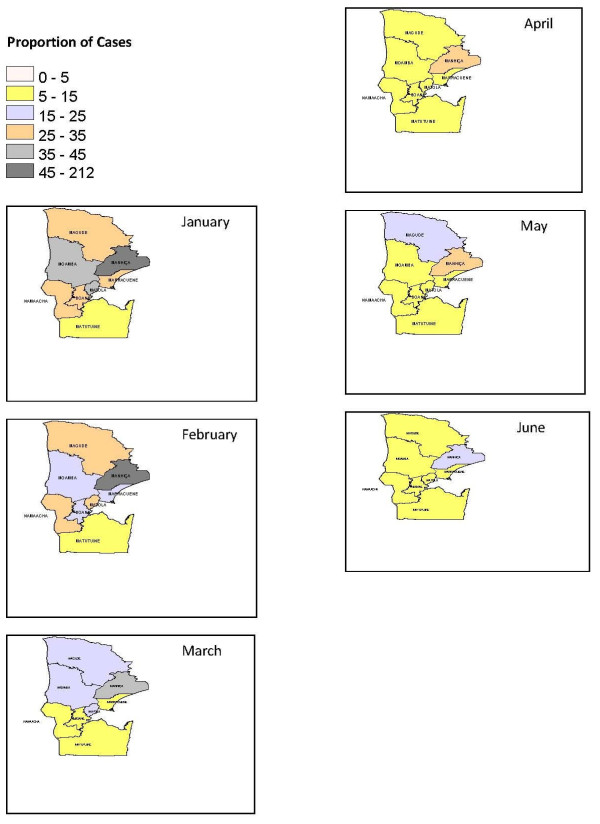
**Estimated RR for months Jan-June**.

Maps of raw malaria rates and posterior Relative Risk (RR) for months July to December are presented as additional files 1 and 2 respectively. Mapped proportions of raw malaria rates and relative risk of malaria incidence were obtained as average of two years for every month in each district of the province. A significant variation in space-time pattern of malaria incidence in the region can be observed in Figure 2 of crude malaria compared to estimated RR, Figure 3.

Specially, raw rates are very high for the most populated districts of Manhiça and Matola. Same pattern is observed in Magude district. Smoothing on the maps of malaria proportions cases is evident in most of the months except for January and February, in districts of Manhiça and Matola. Lowest variations are observed in districts of Matutuine, Moamba and Namaacha. In August we observe a change in smoothing that has lead to an increase of incidence risk in Matutuine district.

Malaria cases are mostly concentrated in months October to March which coincides with wet season in Mozambique. This pattern is also followed by the seasonal variations in climatic conditions of rainfall and humidity. It is observed mainly for coastal districts of Boane, Manhiça, Marracuene and Matola, where in summer (wet) season it is specially humid and rainy. The combination of these covariates brings high probably conditions of mosquitoes breeding and survival leading to increased parasite development [16].

## Discussion

The objective of this study was to analyse the space-time variation of malaria incidence in children less than five years of age in Maputo province. The results show that malaria incidence risk follows a space-time pattern, with some districts at coast-line attaining the highest risk rates. Malaria data aggregation may have played significant role as for the level of incidence risk as well as in relation to its association to environmental predictors. For example [17], found evidence that this situation may inhibit the determination of associations between the levels of malaria incidence and climatic variables in some districts when in most cases these relationships probably existed.

This study, applied Bayesian modelling techniques to analyse patterns of malaria incidence risk considering climatic factors using malaria cases from two years period. To have an understanding of factors associated with probability of development of malaria episodes in children, a Poisson regression model was developed. In order to explain the variation in the response variable, a number of variables are used and they include spatial, fixed, temporal and spatio-temporal terms.

Successes of malaria treatment strategies are tied to the behaviour of patients including mothers and children's caretakers. Treatment-seeking behaviour of health services depends on several factors as, access to health centres, cost of service, level of education, economic status, etc. [18,19]. Although the level of under reporting of diseases episodes was not considered, the study highlights areas of high malaria incidence risk. The conjugated spatio-temporal trend relating district and month has a large influence on the incidence of malaria in the region. It shows that malaria incidence risk varies along each district and for any particular month. The correlated spatial structure was found to be less important. High malaria incidence risk in wet season, particularly for January and February was found in number of other related studies [20-22]. Suitable conditions for malaria transmission are obtained through a combination of humidity and rainfall. However, unexpected results relate to independence of malaria incidence risk in respect to temperature covariates particularly the maximum temperature. While this model does not consider seasonality, minimum and maximum temperature may have influenced the limits of transmission following monthly seasonal grouping in the country. Furthermore, this lack of association between malaria incidence and temperature covariates could indicate that warmer average conditions of at least 28°C were required during months of high transmission [20]. In contrast to studies in [20] and [7], the spatio-temporal model fitted in the former relates to the changes of seasonal incidence in all individuals regions of Zimbabwe; whereas the later is restricted to seasonal analyses of only one district of Mozambique.

Figure 3, shows maps of spatial variation and temporal trends at district level with clusters of high incidence in districts of Manhiça and Matola, for January and February. This suggests that most of the incidence variation might be explained by the individual-level characteristics and also by factors related to inaccessibility to formal health care across the corresponding district. Factors as time constraints, lack and cost of transportation, formal work, competing priorities at home such as child care and food preparation are determinant towards health seeking behaviour [8,9]. Quality of care (e.g. unavailability due to absence of health personnel and long queues) can also discourage households to seek care at health facilities, leading to its increased negative effect [23]. Thus, differences in health care access or even on care quality may induce spatial clustering in health care utilization. Other factor of paramount importance relates to drug resistance [24]. This situation is exacerbated when taking into consideration the susceptibility to malaria created by the HIV infections.

Other reasons for this spatial variation are not certain but could be explained by seasonal transition and the initial waves of annual recurring epidemics [25].

## Conclusions

The knowledge of the incidence of *falciparum *malaria in each epidemiology context is important to the implementation of measures and ideas for treatment and control. This study has shown that malaria transmission in children less than five years of age in period 2007-2008 was stable in most of the districts in Maputo province. The relevance of the study is higher if considering that young children up to five years of age are subjected to the highest risk of malaria infection. Thus, epidemiological studies relating malaria episodes and environmental conditions are of crucial importance to support the need for renewed efforts in environmental management for malaria control in endemic areas. Quantification of malaria incidence variation through the use of robust Bayesian methods is nowadays proven to be of vital importance for monitoring, treatment and control of the disease. This is further enhanced by the possibility of allowing the incidence to vary in spatial and temporal perspective, with the inclusion of different factors particularly climatic predictors. In this study, spatio-temporal patterns of malaria incidence in Maputo province were found to be mainly driven by humidity and rainfall climatic variables.

## Abbreviations

MCMC: Markov chain Monte Carlo; DIC: Deviance information criteria; INAM: National Mozambique National Meteorology Institute; RR: Relative risk; UEM: Universidade Eduardo Mondlane; SIDA: Swedish International Development Cooperation Agency; CI: Confidence interval; SE: Standard error.

## Competing interests

The authors declare that they have no competing interests.

## Authors' contributions

PM was responsible for interpretation of results and manuscript's revision.

OPZ conceived and designed the study and performed: data cleaning, analysis and results interpretation, and manuscript drafting. The authors read and approved the manuscript.

## Supplementary Material

Additional file 1 **Geographical variation in the proportions of malaria cases for months July to December**. Click here for file

Additional file 2 **Geographical variation in the proportions of smoothed malaria cases for months July to December**. Click here for file
