# Supplementary figures and images for: Comparison of infant malaria incidence in districts of Maputo province, Mozambique
Source: Malar J. 2011 Apr 17;10:93. doi: 10.1186/1475-2875-10-93 (PMC3098209; doi:10.1186/1475-2875-10-93)

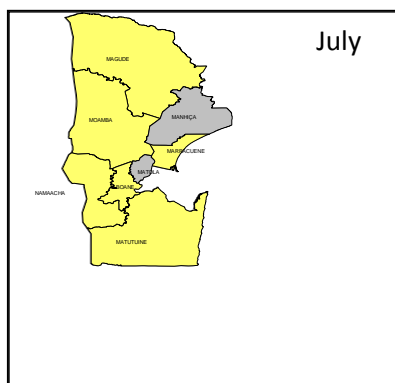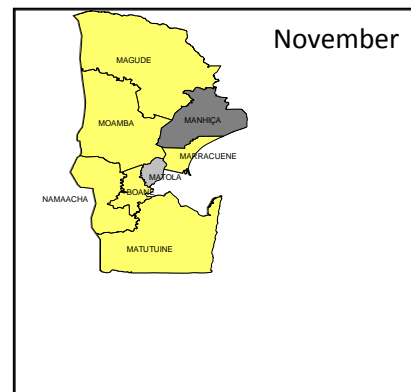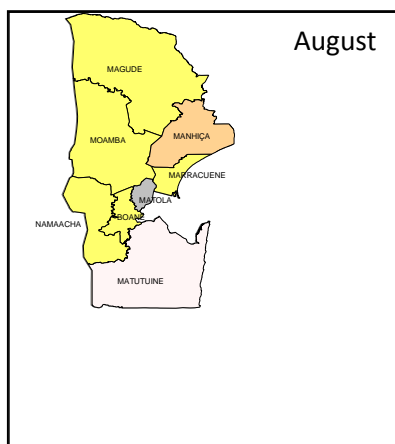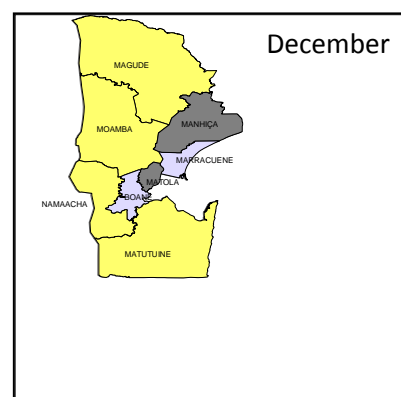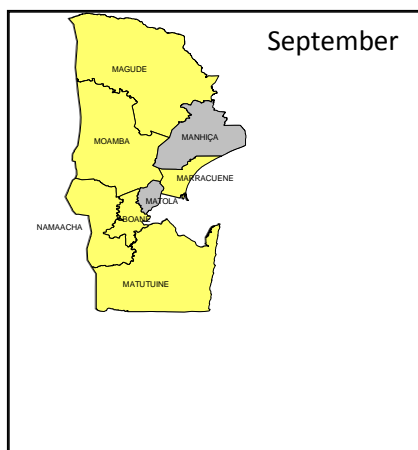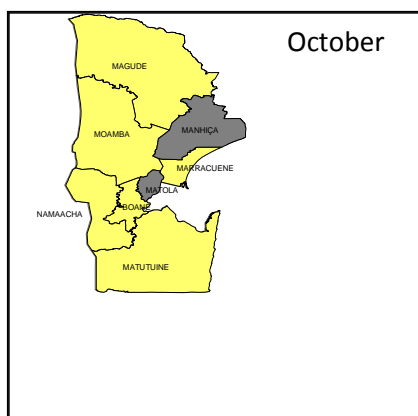

Supplement: Additional file 1 — Geographical variation in the proportions of malaria cases for months July to December. [file 1475-2875-10-93-S1.PDF]

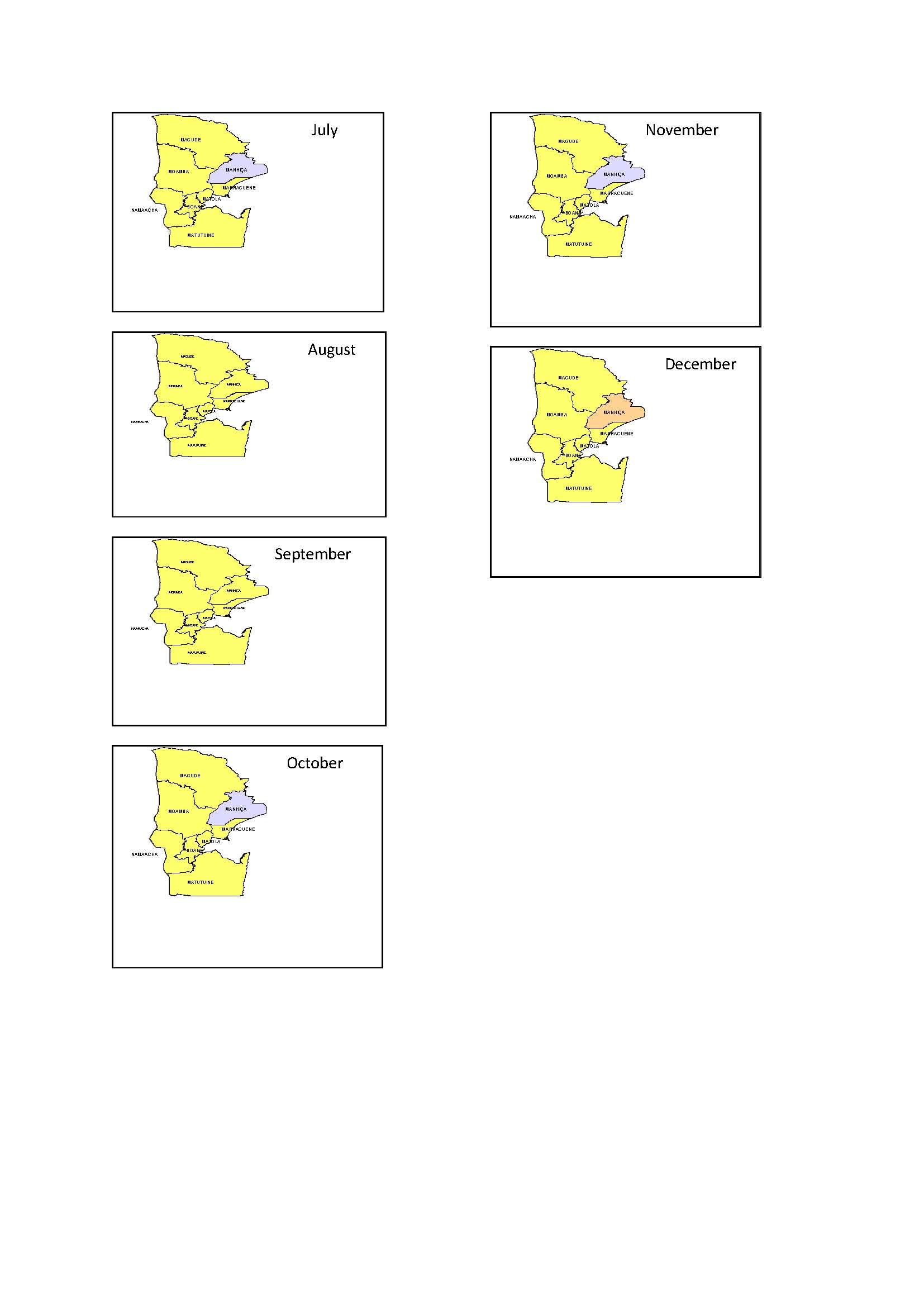

Supplement: Additional file 2 — Geographical variation in the proportions of smoothed malaria cases for months July to December. [file 1475-2875-10-93-S2.JPEG]
